# Clinical Comparison of Outcomes of Early versus Delayed Carotid Artery Stenting for Symptomatic Cerebral Watershed Infarction due to Stenosis of the Proximal Internal Carotid Artery

**DOI:** 10.1155/2016/6241546

**Published:** 2016-11-28

**Authors:** Huakun Liu, Jianfeng Chu, Lei Zhang, Chaolai Liu, Zhongrui Yan, Shengnian Zhou

**Affiliations:** ^1^Department of Neurology, Jining No. 1 People's Hospital, Jining, Shandong 272011, China; ^2^Brain Science Research Institute, Shandong University, No. 107 Wenhuaxi Road, Jinan, Shandong 250012, China; ^3^Department of Neurology, Qilu Hospital of Shandong University, No. 107 Wenhuaxi Road, Jinan, Shandong 250012, China

## Abstract

The aim of this study was to compare the clinical outcomes of early versus delayed carotid artery stenting (CAS) for symptomatic cerebral watershed infarction (sCWI) patients due to stenosis of the proximal internal carotid artery. We retrospectively collected clinical data of those who underwent early or delayed CAS from March 2011 to April 2014. The time of early CAS and delayed CAS was within a week of symptom onset and after four weeks from symptom onset. Clinical data such as second stroke, the National Institutes of Health Stroke Scale (NHISS) score, and modified Rankin Scale (mRS) score and periprocedural complications were collected. The rate of second stroke in early CAS group is lower when compared to that of delayed CAS group. There was no significant difference regarding periprocedural complications in both groups. There was a significant difference regarding mean NHISS score 90 days after CAS in two groups. Early CAS group had a significant better good outcome (mRS score ≤ 2) than delayed CAS group. We suggest early CAS for sCWI due to severe proximal internal carotid artery stenosis as it provides lower rate of second stroke, comparable periprocedural complications, and better functional outcomes compared to that of delayed CAS.

## 1. Introduction

Previous studies have demonstrated that 20% of ischemic strokes are associated with extracranial carotid artery stenosis [1, 2]. Severe stenosis of proximal internal carotid artery can cause ipsilateral cerebral watershed infarction [3]. For patients with carotid artery stenosis, medical therapy and risk factor regulation are considered as first-line treatments. In recent decades, many clinical trials have demonstrated the benefits of carotid revascularization using carotid artery stenting (CAS) or carotid endarterectomy (CEA) in patients with symptomatic carotid stenosis more than 70% [2, 4]. Currently, CAS has become an acceptable treatment methods alternative to the standard CEA. To some patients, CAS may be the optimal treatment option because CEA is contraindicated in such cases due to technical or medical reasons [5]. However, little effort has been given to the study of optimal time management in CAS.

For many years delayed surgical intervention for more than 4 weeks from the onset of symptoms has been conducted in the setting of acute stroke due to the concern for recurrent intraoperative stroke and ischemic to hemorrhagic stroke conversion [6–8]. However, many patients suffered from secondary ischemic stroke during the waiting period of carotid revascularization and thus had poor outcomes [9]. Therefore, in recent years early surgical intervention within 1-2 weeks of symptom onset has been advocated for patients with symptomatic carotid artery stenosis [10, 11]. However, in early stage of symptomatic cerebral watershed infarction (sCWI), CAS remains challenging because patients may suffer from hyperperfusion syndrome or hemorrhagic infarction after revascularization. To the best of our knowledge, there are few data regarding the optimal timing of CAS in sCWI due to proximal internal carotid artery stenosis. The aim of this study was to compare the clinical effect and safety of early versus delayed CAS in patients with sCWI due to proximal internal carotid artery stenosis.

## 2. Patients and Methods

### 2.1. Patients

**Figure 1:**
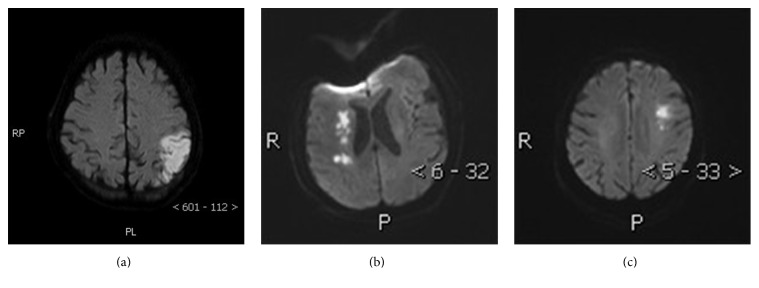
Typical examples of cerebral watershed infarcts in patients with carotid artery stenosis. Diffusion weighted imaging (DWI) showing (a) cortical watershed infarction between the posterior cerebral artery (PCA) and middle cerebral artery (MCA) cortical territories, (b) right-hemisphere internal watershed infarction on diffusion weighted imaging, and (c) cortical watershed infarction between the anterior cerebral artery (ACA) and MCA cortical territories.

The study was approved by the local ethics committee and conducted in accordance with Declaration of Helsinki. All patients gave their written informed consents. We retrospectively collected clinical data of patients with symptomatic cerebral watershed infarction due to stenosis of the proximal internal carotid artery that underwent CAS at our hospital from March 2011 to April 2014. Cerebral watershed infarction was diagnosed by CT or MRI examinations according to the criteria of classic neuropathologic classification as indicated by Momjian-Mayor and Baron [3]. Typical radiological images were shown in Figure 1. The degree of the stenosis of proximal internal carotid artery was used to determine digital subtraction angiography (DSA) according to NASCET criteria.

The inclusion criteria were as follows: (1) 40 ≤ age ≤ 80; (2) patients who had ipsilateral carotid artery stenosis; (3) stenosis of proximal internal carotid artery ≥ 70%; (4) National Institutes of Health Stroke Scale score ≧ 1 and ≤15; (5) patients who can tolerate antiplatelet medications; (6) CAS within one week or after 4 weeks; (7) CAS which is warranted and CEA which is prohibitive, including patients with prohibitive cardiac issues, previous carotid surgery, or prior neck radiation or refusing for CEA.

The exclusion criteria were as follows: (1) patients aged < 40 or >80; (2) National Institutes of Health Stroke Scale score > 15; (3) patients with ipsilateral carotid artery occlusion; (4) patients with other types of cerebral infarction; (5) patients with stenosis of proximal internal carotid artery < 70%.

Demographic and clinical characteristics of patients such as age, gender, hypertension, hyperlipidemia, diabetes mellitus, coronary artery disease, and smoking status in two groups were collected. Patients receiving CAS within one week of symptom onset were classified in the early CAS group and patients receiving CAS after 4 weeks from sCWI onset were classified in the delayed CAS group. The reason that we focused on the patients with cerebral watershed infarction was due to the fact that this specific type of cerebral infraction has relatively weak blood flow perfusion within the watershed regions and thus may reduce the risk of hyperperfusion and cerebral hemorrhage after early CAS. Another reason is that watershed regions are more likely to develop infraction penumbra which may lead to more cerebral cell functional recovery and improve prognosis after early CAS.

### 2.2. Intervention Protocols

**Figure 2:**
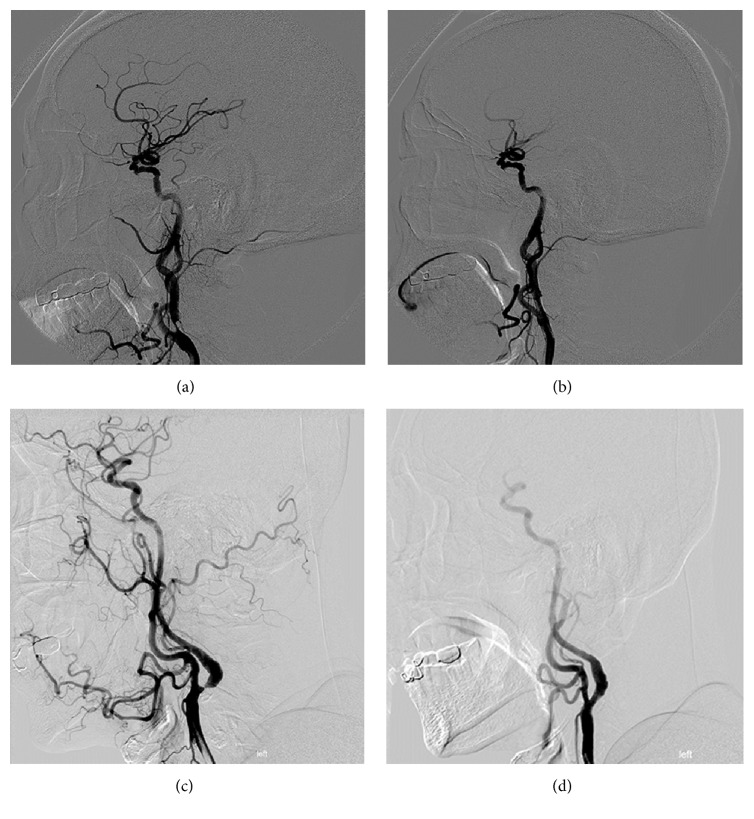
A closed-cell stent was used in a straight stenotic segment ((a)-(b)). An open-cell stent was used in a curved stenotic segment ((c)-(d)).

For patients in early CAS group, daily therapy of aspirin (300 mg) and of clopidogrel (300 mg) was implemented prior to CAS. For patients in delayed CAS group, daily therapy of aspirin (100 mg) and of clopidogrel (75 mg) was implemented seven days prior to the CAS treatment. CAS was performed under local anesthesia by two experienced neurointerventionists. First, a 8F arterial sheath was punctured into the femoral artery using Seldinger technique. A 8F guiding catheter was placed into the common carotid artery at a distance of 3-4 cm from the stenosis. Second, embolic protection device (Filter Wire EZ System, Boston Scientific Corporation, USA) was delivered through the stenotic segment to 4–6 cm distal to the stenosis and opened. Thirdly, predilation was performed using a 4-5 mm balloon (Sterling Monorail, Boston Scientific Corporation, USA). Fourthly, a closed-cell stent (Wallstent Carotid Stent System, Boston Scientific Corporation, USA) was positioned in a straight stenotic segment or an open-cell stent (Acculink Carotid Stent System, Abbott Vascular, USA, or Wallstent Carotid Stent System, Boston Scientific Corporation, USA) was positioned in a curved stenotic segment (Figure 2). If the stenotic segment was not fully dilated after stent deployment, postdilation was performed using a 4 or 5 mm balloon (Sterling Monorail, Boston Scientific Corporation, USA). Finally, the embolic protection device was removed and punctured femoral artery was sutured.

During the periprocedural period, atropine and dopamine were used if patients underwent descent of heart rate or blood pressure. After the procedure, ambulatory blood pressure was monitored for 48 h. Systolic blood pressure was controlled within the level about 80–90% of preendovascular treatment. After the procedure, all patients received aspirin (100 mg/d) and clopidogrel (75 mg/d) for the first 3 months and aspirin (100 mg/d) thereafter.

### 2.3. Clinical Evaluation and Follow-Up

All patients were advised to come back and see their doctor if there existed cerebral infarction or cerebral hemorrhage after discharge and however come back to the hospital for a check 90 days after discharge from the hospital if there did not exist cerebrovascular disease. The following data such as occurrence of second stroke, the National Institutes of Health Stroke Scale (NHISS) score, and modified Rankin Scale (mRS) score at admission and 90 days after DAS and periprocedural complications were collected.

In this study, mRs scores of 0, 1, or 2 were defined as good outcome and 3 to 6 as poor outcomes. Second stroke was defined as recurrent neurologic symptoms due to ipsilateral carotid artery stenosis during the CAS waiting period. Periprocedural complications were documented including any periprocedural ischemic stroke, hyperperfusion syndrome, cerebral hemorrhage, or death within 30 days after CAS [4]. Ischemic stroke was defined as a rapidly developing clinical syndrome of focal disturbance of cerebral function lasting more than 24 h or leading to death with no apparent cause other than that of vascular origin [12]. Hyperperfusion syndrome was defined as a group of symptoms after revascularization with severe headache, epilepsy, disturbance of consciousness, and focal neurological deficits as the main clinical manifestations [13].

### 2.4. Statistical Analysis

Data analysis was performed by using SPSS software, version 13.0 (SPSS Inc., Chicago, IL). Measurement data was expressed as mean ± standard deviation (means ± SD) and compared using Student *t*-test. Count data was expressed as number or percentage and compared using chi-square test. A *P* value of less than 0.05 was considered as statistically significant.

## 3. Results

### 3.1. Baseline Data

A total of 120 patients with symptomatic cerebral watershed infarction due to stenosis of the proximal internal carotid artery treated with CAS were included in this study. Of the 120 patients, 63 cases were treated with CAS within a week of symptom onset (early CAS group) while the remaining 57 cases were treated with CAS after 4 weeks from symptom onset (delayed CAS group). The mean age of patients in early CAS group and delayed CAS group was 64.03 ± 3.74 years and 64.12 ± 3.38 years, respectively. The mean of NIHSS score at admission was 8.52 ± 2.46 in early CAS group and 7.84 ± 2.64 in delayed CAS group. Patients whose mRS scores ≤ 2 at admission were 24 (38%) in early CAS group and 17 (29%) in delayed CAS group. The mean stenosis of patients in early CAS group and delayed CAS group was 82.67 ± 7.70% and 82.51 ± 7.24%, respectively. There was no statistically significant difference with regard to the age, sex, medical history, NHISS score at admission, number of patients whose mRS score ≤ 2 at admission, and mean stenosis between the early and delayed CAS groups (Table 1).

### 3.2. Periprocedural Outcomes

All CAS was successfully completed in both groups. The technical successful rate was 100%. Two patients (3.2%) in early CAS group experienced second stroke presenting with aggravated hemiparalysis during the waiting period of carotid revascularization. Nine (15.8%) patients in delayed CAS group experienced second stroke presenting with aggravated neurologic symptoms during the waiting period of carotid revascularization. The rate of second stroke in early CAS group is lower when compared to that of delayed CAS group (*P* = 0.032) (Table 2). During the periprocedural period, 51 (81.0%) patients in early CAS group and 43 (75.4%) patients in delayed CAS group suffered from descent of heart rate or blood pressure (*P* = 0.464) (Table 2). These patients restored to normal following atropine and dopamine therapy.

With regard to the periprocedural complications, there were 3 (4.8%) patients in early CAS group and 3 (5.3%) patients in delayed CAS group with no statistical significant difference (*P* = 0.900) (Table 2). Among the three patients in early CAS group, one patient suffered from ipsilateral cerebral hemorrhage on the fourth day after CAS, which was completely absorbed 2 weeks after CAS due to the fact that the hemorrhage volume was less than 5 milliliters. The remaining two patients suffered periprocedural ipsilateral stroke and had poor outcomes in 90 days, while among the three patients in delayed CAS group, one patient suffered ipsilateral cerebral hyperperfusion after CAS and his symptoms were greatly improved after drug treatment. The remaining two patients suffered periprocedural ipsilateral stroke which aggravated their neurologic symptoms. No death was noted in both groups. No acute cerebrovascular disease was noted in both groups within 90 days of hospital discharge.

### 3.3. Functional Outcomes

The mean NHISS score 90 days after CAS was 2.70 ± 1.46 in early CAS group and 3.51 ± 1.71 in delayed CAS group, respectively (Table 3), with a statistical significant difference (*P* = 0.006). The *D*-value of postoperative and preoperative NHISS score in two groups was −5.83 ± 2.14 and −4.33 ± 2.07, respectively (*P* < 0.05) (Table 3). Good outcomes (mRS score ≤ 2) was obtained in 50 patients (79%) in early CAS group and in 35 (61%) patients in delayed CAS group. The rate of good outcomes in early CAS group was significant higher than that in delayed CAS group (79% versus 61%, *P* = 0.031).

## 4. Discussion

Traditional concepts suggest that new cerebral infarction should be treated after four weeks from symptom onset due to the fact that it can reduce the risk of postoperative bleeding or cerebral hyperperfusion syndrome [6–8]. However, patients may experience secondary stroke during the waiting period of carotid revascularization, thus aggravating the neurologic impairment [7, 9, 14]. Furthermore, patients may be unable to achieve an ideal recovery due to the ipsilateral severe carotid artery stenosis [15]. In recent years early intervention within 1-2 weeks of symptom onset has been advocated [10, 11]. Several studies assessed safety data on early CAS after symptom onsets and however achieved conflicting results [16–20]. Up till now, the optimal timing of CAS for patients with symptomatic cerebral infarction remains unclear. In this study, we compared the clinical outcomes of early versus delayed CAS for sCWI patients due to stenosis of the proximal internal carotid artery. We found that early CAS can be selected as a suitable approach for sCWI resulting from severe proximal internal carotid artery stenosis because it can provide lower rate of second stroke, comparable periprocedural complications, and better functional outcomes compared to delayed CAS. Our study provides a reference for application of early CAS in sCWI due to severe proximal internal carotid artery stenosis.

After reviewing the previous literatures [21, 22], we found that most of trials supporting delayed intervention in cerebral infarction did not screen patients according to imaging characteristics and strictly control postoperative blood pressure and thus had a higher rate of postoperative hyperperfusion and bleeding in ipsilateral brain tissues. In this study, after strictly screening for symptomatic cerebral infarction patients with NHISS score ≤ 15 and controlling postoperative blood pressure, we found that early stenting had a comparable periprocedural complications compared to that of delayed stenting. Therefore, after strictly screening for infarction type and postoperative control of blood pressure, early CAS is safe in sCWI.

Previous studies reported that secondary stroke is prone to develop during the waiting period of carotid revascularization for patients with recent cerebral infarction and concomitant ipsilateral severe carotid artery stenosis [7, 9, 14]. Ferrero et al. reported that the early risk of secondary stroke after transient ischemic attack (TIA)/stroke is approximate 5–10% at 1 week and 10–20% at 3 months [23]. In this study, we also found that the delayed CAS group had a higher rate of second rate stroke compared to that of early CAS group (12.3% versus 3.2%). To reduce the risk of secondary stroke in patients with sCWI and concomitant ipsilateral severe carotid artery stenosis, early stenting should be advocated.

Before data analysis, we hypothesized that early relieving carotid artery stenosis in sCWI and increasing blood perfusion of ipsilateral brain tissues may promote neurological function recovery. Thus we analyzed the functional outcomes from the following three perspectives. Firstly, we compared the mean NHISS score 90 days after CAS in two groups and found that the early CAS group had a significant lower NHISS score than that of delayed CAS group. Furthermore, we compared the variation value of postoperative and preoperative NHISS score and found that early CAS group had a significant higher declined range regarding the *D* value of NHISS score than that of the delayed CAS group. These findings suggest that early CAS had a higher advantage in reducing the NHISS score than that of the delayed CAS group. Moreover, we compared the rate of good outcomes (mRS score ≤ 2) 90 days after CAS in two groups and found that early CAS group had a higher rate of good outcomes than that in delayed CAS group (79% versus 61%). These all aforementioned findings suggest that early stenting has a higher advantage in improving the prognosis of patients with sCWI than that of delayed CAS group.

## 5. Conclusions

We suggest early CAS as a suitable approach for sCWI due to severe proximal internal carotid artery stenosis as such a method provides lower rate of second stroke, comparable periprocedural complications, and better functional outcomes compared to that of delayed CAS after strictly screening for infarction type and postoperative control of blood pressure. Further studies with a larger number of patients and longer time of follow-up are warranted.

## Figures and Tables

**Table 1:** Baseline data of 120 patients with symptomatic cerebral watershed infarction.

	Early CAS group (*n* = 63)	Delayed CAS group (*n* = 57)	*P* value
Age (years)	64.03 ± 3.74	64.12 ± 3.38	0.89^a^
Gender			0.84^b^
Male (*n*)	32	31	
Female (*n*)	31	27	
Medical history			
Hypertension (*n*)	40	33	0.53^b^
Hyperlipidemia (*n*)	36	34	0.78^b^
Diabetes mellitus (*n*)	15	17	0.46^b^
CAD (*n*)	18	16	0.95^b^
Smoking (*n*)	24	21	0.89^b^
NHISS score at admission	8.52 ± 2.46	7.84 ± 2.64	0.15^a^
mRS score ≤ 2 at admission, *n* (%)	24 (38%)	17 (29%)	0.41^b^
Mean stenosis (%)	82.67 ± 7.70	82.51 ± 7.24	0.91^a^

Data was expressed as means ± SD; number or percentage was expressed as appropriate. CAD, coronary artery disease; NHISS score, National Institutes of Health Stroke Scale score; mRS score, modified Rankin Scale score.

^a^ *t*-test.

^b^Chi-square test.

**Table 2:** Periprocedural outcomes in two groups.

	Early CAS group (*n* = 63)	Delayed CAS group (*n* = 57)	*P* value
Second stroke	2 (3.2%)	9 (15.8%)	*P* = 0.024^a^
Descent of heart rate or blood pressure	51 (81.0%)	43 (75.4%)	*P* = 0.464^a^
Periprocedural complications	3 (4.8%)	3 (5.3%)	*P* = 0.900^a^
Cerebral hemorrhage	1 (1.6%)	0	
Periprocedural stroke	2 (3.2%)	2 (3.2%)	
Hyperperfusion syndrome	0	1 (1.7%)	
Cerebrovascular disease	0	0	
Death	0	0	

^a^Chi-square statistics.

**Table 3:** Functional outcome 90 days after CAS in both groups.

	Early CAS Group	Delayed CAS Group	*P* value
NHISS score 90 days after CAS	2.70 ± 1.46	3.51 ± 1.71	0.006^a^
*D*-value of NHISS score	−5.83 ± 2.14	−4.33 ± 2.07	0.000^a^
mRS score ≤ 2 90 days after CAS, *n* (%)	50 (79%)	35 (61%)	0.031^b^

NHISS score, National Institutes of Health Stroke Scale score. *D*-value, the difference between mean NHISS score 90 days after CAS and mean NHISS score at admission. mRS score, modified Rankin Scale score.

^a^Independent sample *t*-test.

^b^Chi-square statistics.
